# miR-191-5p suppresses PRRSV replication by targeting porcine EGFR to enhance interferon signaling

**DOI:** 10.3389/fmicb.2024.1473504

**Published:** 2024-10-14

**Authors:** Yu Pan, Lin Zhang, Wenjie Ma, Yassein M. Ibrahim, Wenli Zhang, Mengjie Wang, Xinrong Wang, Yunfei Xu, Caixia Gao, Hongyan Chen, He Zhang, Changyou Xia, Yue Wang

**Affiliations:** ^1^State Key Laboratory for Animal Disease Control and Prevention Heilongjiang Provincial Key Laboratory of Laboratory Animal and Comparative Medicine, Harbin Veterinary Research Institute, Chinese Academy of Agricultural Sciences, Harbin, China; ^2^National Center of Technology Innovation for Pigs, Chongqing Academy of Animal Science, Chongqing, China; ^3^College of Veterinary Medicine, Southwest University, Chongqing, China

**Keywords:** porcine reproductive and respiratory syndrome virus, miR-191-5p, porcine epidermal growth factor receptor, signal transducer and activator of transcription 3, IFN-I

## Abstract

Porcine reproductive and respiratory syndrome virus (PRRSV) is a major thread to the global swine industry, lack of effective control strategies. This study explores the regulatory role of a small non-coding RNA, miR-191-5p, in PRRSV infection. We observed that miR-191-5p significantly inhibits PRRSV in porcine alveolar macrophages (PAMs), contrasting with negligible effects in MARC-145 and HEK293-CD163 cells, suggesting a cell-specific antiviral effect. Further investigation unveiled that miR-191-5p directly targets the porcine epidermal growth factor receptor (EGFR), whose overexpression or EGF-induced activation suppresses type I interferon (IFN-I) signaling, promoting PRRSV replication. In contrast, siRNA-or miR-191-5p-induced EGFR downregulation or EGFR inhibitor boosts IFN-I signaling, reducing viral replication. Notably, this miRNA alleviates the suppressive effect of EGF on IFN-I signaling, underscoring its regulatory function. Further investigation revealed interconnections among miR-191-5p, EGFR and signal transducer and activator of transcription 3 (STAT3). Modulation of STAT3 activity influenced IFN-I signaling and PRRSV replication, with STAT3 knockdown countering EGFR activation-induced virus replication. Combination inhibition of STAT3 and miR-191-5p suggests that STAT3 acts downstream in EGFR’s antiviral response. Furthermore, miR-191-5p’s broad efficacy in restricting various PRRSV strains in PAMs was identified. Collectively, these findings elucidate a novel mechanism of miR-191-5p in activating host IFN-I signaling to inhibit PRRSV replication, highlighting its potential in therapeutic applications against PRRSV.

## 1 Introduction

Porcine reproductive and respiratory syndrome virus (PRRSV) is a major pathogen threatening the global swine industry, resulting to significant economic losses due to reproductive disorders in sows and severe respiratory diseases in piglets (Zhou and Yang, 2010; Lunney et al., 2016). PRRSV is a single-stranded RNA enveloped virus of approximately 15.4 kb genome, containing at least 9 opening reading frames (ORFs). PRRVS belongs to the family *Arteriviridae* of the order *Nidovirales*, which is classified into two species, *Betaarterivirus suid 1* (referred as PRRSV-1, the European type) and *Betaarterivirus suid 2* (referred as PRRSV-2, the North American type) (Xu et al., 2021; Pei et al., 2023). In 2006, highly pathogenic PRRSV (HP-PRRSV) was reported in China, characterized by high fever high, morbidity, and mortality in infected pigs (Li et al., 2007). Since then, HP-PRRSV has become one of the most predominant strains in China (Liu et al., 2022; Li et al., 2024). Despite the development and use of various commercial PRRSV vaccines, the complexity and rapid mutation propensity of PRRSV have led to poor immunological protection in the clinic settings (Corzo et al., 2010; Rathkjen and Dall, 2017). Furthermore, PRRSV-induced immunosuppression, long-term carriage of the virus, and antibody-dependent enhancement pose significant challenges for novel vaccine design (Rahe and Murtaugh, 2017; Cai et al., 2023; Zhang H. et al., 2023). Therefore, it is an essential need to explore additional strategies against PRRSV infection. As a promising class of antiviral agents, miRNA-based therapies have been demonstrated in the clinical application (Janssen et al., 2013). Identifying miRNAs with antagonistic effects on PRRSV is crucial for the prevention, control and intervention of the virus.

The miRNA, a group of small non-coding RNAs of 19–24 nucleotides, mainly exert their regulatory effects through binding to the 3′-untranslated region (3′UTR) of target mRNAs by their seed sequences (Eulalio et al., 2008; Wilczynska and Bushell, 2015; O'Brien et al., 2018). This binding process is mediated by Argonaute proteins (AGOs), which are crucial for the miRNA’s function (Gregory et al., 2005; MacRae et al., 2008). Extensive studies have shown the significant role of miRNAs in both host immunity responses and viral infection (Katopodis et al., 2022; Pandita et al., 2023). In the context of innate immunity and the IFN-I signaling pathways, specific miRNAs have been identified to modulate the antiviral response. For instance, miR-466l targets IFN-*α* to reduce the antiviral response (Li et al., 2012), while miR-155 enhances this process by promoting IFN-I signaling through suppressor of cytokine signaling 1 (SOCS1) (Ye et al., 2016). In the realm of virology, miR-122 has been shown to directly target the Hepatitis C Virus (HCV) genome, thereby enhancing its stability and translation efficiency. Consequently, therapeutic interventions targeting miR-122 have the potential to inhibit HCV replication (Janssen et al., 2013). Additionally, other miRNAs, including miR-486-5p, mir-487b-5p, miR-654, and let-7c, have been implicated in the degradation of viral RNA and the inhibition of viral replication (Song et al., 2010; Ma et al., 2012; Peng et al., 2018). Viruses are also known to exploit host cellular miRNAs to their advantage. For example, miR-27b-3p can suppress host proteins like SOCS6, which in turn influences transmissible gastroenteritis virus (TGEV) replication (Wang et al., 2022).

Researchers have dedicated nearly a decade to studying miRNAs in pigs and have identified over 800 distinct types. Some of these, like miR-181 and miR-320, have been validated for their inhibitory effects on PRRSV infection, as documented in studies (Guo et al., 2013; Gao et al., 2024). However, there are still many other pig miRNAs remain to be fully elucidated. In this study, we have focused our efforts on a particular miRNA, miR-191-5p, aiming to uncover its regulatory role in the context of PRRSV infection. We anticipate that our research will shed light on its potential influence and contribute to a deeper understanding of PRRSV-host interactions.

## 2 Materials and methods

### 2.1 Animal, cells and viruses

Primary porcine alveolar macrophages (PAMs) were isolated from lungs of 5-week-old specific-pathogen-free (SPF) pigs and cultured in RPMI-1640 medium (Gibco, USA), supplemented with 10% fetal bovine serum (FBS, Hyclone, USA), 1% penicillin, and 1% streptomycin (Beyotime, China). HEK293T (ATCC CRL-3216) and MARC-145 (ATCC CRL-12231) were grown in Dulbecco’s modified Eagle’s medium (DMEM, Gibco, USA), supplemented with FBS. The HP-PRRSV strain HuN4 (GenBank no. EF635006) was propagated and titrated in MARC-145 cells.

### 2.2 Lentiviral preparation

The lentiviral vector pLVX-IRES-ZsGreen1, along with psPAX2 and pMD2.G were transfected into HEK293T at a ratio of 3:2:1. The viral supernatants were collected at 48 and 72 h after transfection. The cell supernatants were centrifuged at 4,000 × *g* for 5 min at 4°C and filtered through a 0.45 μm filter (Millipore). HEK293T cells cultured in 96-well-plate with 5 × 10^4^ cells per well, were infected with the lentivirus after 10-fold dilution with 6 μg/mL polybrene. After 3–4 days, the number of fluorescent cells in the last two fluorescent dilution gradients were counted with the following equation. The titer (TU/mL) = (X + Y × 10) × factor of dilution × 10.

### 2.3 RNA oligo transfection and lentiviral transduction of PAMs

RNA oligos including miRNA inhibitor, miRNA mimic, and small interfering RNA (siRNA) were synthesized by Genepharma (Suzhou, China). The RNA oligo sequences are listed in Table 1. PAMs were cultured on poly-L-lysine (Sigma) treated plates. After cells adhesion, the siRNA, miRNA mimic miRNA inhibitor, 3′-biotinylated miRNA mimic and the corresponding negative control at the concentration of 100 nM were transfected into PAMs for 24 h using lipofectamine RNAiMAX reagent (Invitrogen, USA) according to the manufacturer’s instructions. After applying various specified treatments, cell monolayers were exposed to lentivirus at a multiplicity of infection (MOI) of 5. Following 10 h post-infection (hpi), the monolayers were washed and then incubated further in fresh culture media. Subsequently, the cells were collected for qPCR or Western blotting analysis, and the supernatants were collected for virus titration.

**Table 1:** Small RNA sequences used in this study.

RNA oligo name	Sequence (positive strand) (5′-3′)
si-EGFR	GUUUGUAACGGGAUAGGGATT
si-STAT3	GUCAGAUUGCUGGUCAAAUTT
si/miR-Negative Control	UUCUCCGAACGUGUCACGUTT
miR-191-5p mimic	CAACGGAAUCCCAAAAGCAGCUG
miR-novel-029 mimic	UUCGAAUCACGUCGGGGU
microRNA inhibitor NC	CAGUACUUUUGUGUAGUACAA
miR-191-5p inhibitor	CAGCUGCUUUUGGGAUUCCGUUG
miR-novel-029 inhibitor	ACCCCGACGUGAUUCGAA

### 2.4 RNA extraction and quantitative PCR (qPCR)

Total RNA was extracted from PAMs infected with PRRSV at 0.1 MOI, using RNA extraction kits (BioFlux, China), and reverse transcribed into cDNA using PrimeScript RT reagent Kit with gDNA Eraser by random primers (Takara, Japan) according to the manufacturer’s instructions. The RNA oligo sequences are listed in Table 2. The qPCR was carried out in a QuantStudio 5 system (Applied Biosystems) using SYBR premix Ex Taq (Lablead, China). Fold changes were determined using the cycle threshold (ΔΔCT) method.

**Table 2:** Primers used for relative quantitative RT-PCR.

Primer name	Sequence (5′–3′)
HuN4-ORF7-F	AGATCATCGCCCAACAAAACC
HuN4-ORF7-R	GACACAATTGCCGCTCACTAGG
MLV-ORF7-F	AGATCATCGCTCAGCAAAACC
MLV-ORF7-R	GACACAATTGCCGCTCACTAGG
Ch-1a-ORF7-F	AGATCATCGCCCAACAAAACC
Ch-1a-ORF7-R	GACACAATTGCCGCTCACTAGG
JXA1-ORF7-F	AGATCATCGCCCAGCAAAACC
JXA1-ORF7-R	GACACAATTGCCGCTCACTAGG
Porcine-β-actin-F	CTTCCTGGGCATGGAGTCC
Porcine-β-actin-R	GGCGCGATGATCTTGATCTTC
Human-β-actin-F	CCTTCCTGGGCATGGAGTCCTG
Human-β-actin-R	GGAGCAATGATCTTGATCTTC
Monkey-β-actin-F	AGGCTCTCTTCCAACCTTCCTT
Monkey-β-actin-R	CGTACAGGTCTTTACGGATGTCCA
Porcine-IFN-β-F	GCTAACAAGTGCATCCTCCAAA
Porcine-IFN-β-R	CCAGGAGCTTCTGACATGCCA
Porcine-ISG15-F	GATGCTGGGAGGCAAGGA
Porcine-ISG15-R	CAGGATGCTCAGTGGGTCTCT
Porcine-MxA-F	CACTGCTTTGATACAAGGAGAGG
Porcine-MxA-R	GCACTCCATCTGCAGAACTCAT
Porcine-STAT3-F	CTTGCCAGTCGTGGTCATCT
Porcine-STAT3-R	CACTTGATCCCACGTTCCGA
Porcine-EGFR-F	AGGACGAAGCAACATGGTCA
Porcine-EGFR-R	TGCATAGCACAGGTTTCGGT

### 2.5 Immunofluorescence assay

Immunofluorescence assay (IFA) was performed as previously described with slight modifications (13). Briefly, PAM monolayers were treated with oligo RNA and PRRSV. Subsequently, the PAMs were fixed with 4% paraformaldehyde for 30 min and permeabilized with 0.1% Triton-X100 for 10 min. The monolayer cells were blocked with 2% bovine serum albumin (BSA) after washed twice with PBS, subsequently incubated with mouse anti-PRRSV N protein monoclonal antibody (1:1000) for 2 h. After three washes with PBS, the PAMs were incubated with Alexa Fluor 488-conjugated goat anti-mouse IgG as secondary antibody for 45 min. After three additional washes, PAMs nuclei were stained with DAPI for 10 min at 37°C. Finally, the cells were visualized under an inverted fluorescence microscope equipped with a camera (ZEISS, Jena, Germany).

### 2.6 Western blotting

Western blot analysis was done as previously described with slight modification (Zhang et al., 2021). PAMs were inoculated with PRRSV or mock at a MOI of 0.1 for 24 h. The Cells were lysed in radioimmunoprecipitation assay (RIPA) buffer (HaiGene, China) containing protease and phosphatase inhibitor cocktail (Beyotime, China). The cell lysates were separated by SDS-PAGE and transferred to polyvinylidene difluoride (PVDF) membranes (Merck Millipore, USA). The membranes were blocked in 5% skim milk and incubated with the indicated primary and secondary antibodies. The mouse anti-PRRSV nucleocapsid (N) protein mAb (prepared in our lab) and the mouse anti-*β*-actin mAb (A2228, Sigma) were used as primary antibodies. The IRDye 680 and The IRDye 800 conjugated goat anti mouse IgG (Li-Cor Biosciences) were used as secondary antibodies. Finally, the membranes were scanned with Odyssey infrared imaging system (Li-Cor Biosciences, USA).

### 2.7 miRNA pulldown

The miRNA pulldown was performed according to the procedures described by JoVE with slight modification (Dash et al., 2018). PAMs were seeded in poly-L-lysine (Sigma) treated 6-wells plates at a density of 2 × 10^6^ cells/well. After cells adhered, the 3′-biotinylated miR-191-5p mimic (100 nM) and corresponding negative control were transfected into PAMs for 36 h using lipofectamine RNAiMAX reagent (Invitrogen, USA) according to the manufacturer’s instructions. Subsequently, PAMs were harvested by gentle scraping with a cell scraper and centrifuged at 1,500 × *g* for 5 min. The cell pellet was lysed in 1050 μL cell lysis buffer (150 mM NaCl, 25 mM Tris-Cl, pH-7.5, 5 mM DTT, 0.5% IGEPAL, 1000 U/mL recombinant RNase inhibitor (RRI), 1 × protease inhibitor) for 30 min on ice. After centrifugation at 16,000 × *g* for 5 min, 50 μL of the clear cell lysate supernatant was transferred to a 1.5 mL nuclease-free microfuge tube as input, while the remaining lysates were transferred to another nuclease-free microfuge tube. Streptavidin Magnetic beads (20 μL) were added to the lysates and incubated on a nutating mixer for 4 h at 4°C. The magnetic beads were then washed five times with ice-cold complete pull-down wash buffer (10 mM KCl, 1.5 mM MgCl_2_, 10 mM Tris-Cl (pH 7.5), 5 mM DTT, 1 M NaCl, 0.5% IGEPAL, 100 U/mL RRI, and 1 × protease inhibitor cocktail) and finally diluted with 100 μL of nuclease-free water. The subsequent steps involved RNA extraction and qPCR as described above.

### 2.8 Dual-luciferase reporter assay

HEK293T cells were transfected with either miR-191-5p mimic or negative control, along with pmirGLO-EGFR 3’UTR wild-type or mutant-type reporter plasmid using Lipofectamine 2000 (Invitrogen, USA) for 10 h. The cells medium was replaced with 2% FBS medium and the cells were incubated for an additional 36 h. Subsequently, the cells were lysed with passive lysis buffer, and firefly and renilla luciferase activities were measured using multimode microplate reader and the dual luciferase reporter assay system (Promega), according to the manufacturer’s instructions. Transfection efficiency was normalized using the firefly luciferase activity relative to the renilla luciferase activity. For IFN-*β* promoter activity assay, reporter plasmids (pIFN-β-promoter-Fluc or Pgl3-basical) and pTK-Rluc (0.01 μg), were co-transfected with or without the indicated expression plasmids, using Lipofectamine 2000. Subsequent procedures followed those outlined previously.

### 2.9 Statistical analysis

All data were analyzed for significance using t-tests in GraphPad Prism (GraphPad Software, Inc), and were considered statistically significant at *p* < 0.05. All experimental data were presented as the mean ± standard deviations (SD) and were replicated at least three times.

## 3 Results

### 3.1 miR-191-5p inhibits PRRSV infection

**Figure 1:**
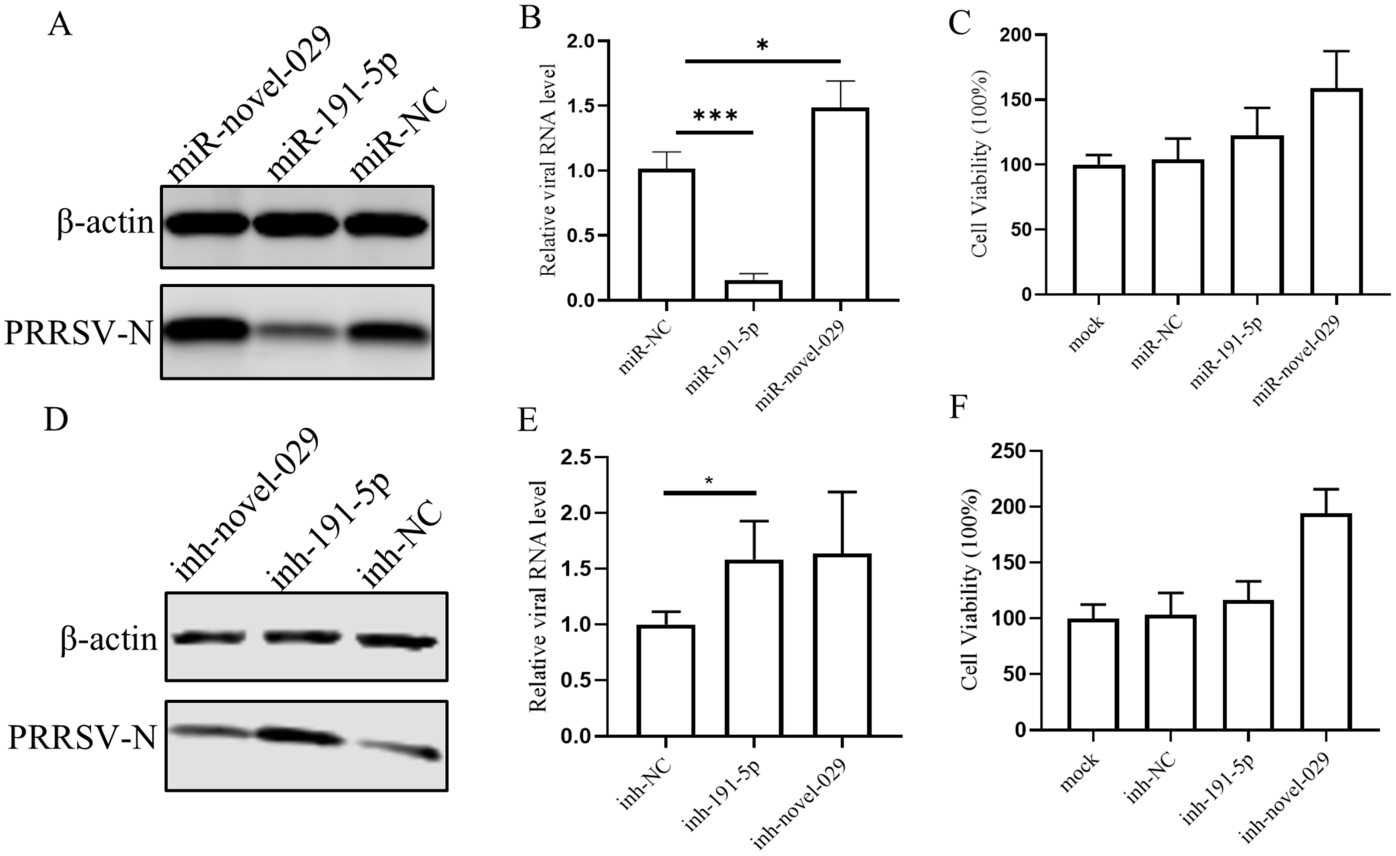
miR-191-5p inhibits PRRSV infection. **(A,B)** miR-191-5p mimic represses PRRSV infection. Primary PAMs were transfected with the mimic of miR-191-5p, miR-novel-029 or negative control (NC) at the concentration of 100 nM for 24 h, followed by inoculation with HuN4 at MOI of 0.1 for an additional 24 h. Cell lysates were subjected to western blot to detect PRRSV-N protein expression **(A)**. Relative viral RNA level was determined by qPCR **(B)**. **(C)** Transfection of miRNAs’ mimic does not affect cell viability. A CCK8 assay was performed on PAMs transfected with the indicated miRNAs mimics or NC for 72 h. **(D,E)** miR-191-5p inhibitor promotes PRRSV infection. PAMs were transfected with miRNA inhibitor or inhibitor-NC for 24 h, and cells were then inoculated with HuN4 at MOI of 0.1 for 24 h. Cell lysates were subjected to western blot to detect PRRSV-N protein expression **(D)**. Relative viral RNA level was quantified by qPCR **(E)**. **(F)** Transfection of miRNAs’ inhibitor does not affect cell viability. A CCK8 assay was performed on PAMs transfected with indicated miRNAs inhibitors or NC for 72 h. Data are presented as the mean ± SD. ^∗^*p* < 0.05; ^∗∗^*p* < 0.01; ^∗∗∗^*p* < 0.001.

In our previous study, we reported a database of miRNAs associated with PRRSV infection in PAMs (Zhang et al., 2021). Expanding upon this groundwork, we here delved deeper into the roles of two specific miRNAs: miR-191-5p and a newly discovered miRNA, miR-novel-029. To assess their effects on PRRSV replication, PAMs were transfected with their mimics and followed by PRRSV infection. The results showed that miR-191-5p significantly reduced PRRSV infection, as indicated by decreased levels of viral RNA and N protein expression. In contrast, miR-novel-029 appeared to enhance virus infection, when compared with the negative control (Figures 1A,B). We then proceeded to test the effects of inhibitors for each miRNA on PAMs. Western blot analysis showed that the inhibitor for miR-191-5p resulted in an increase in PRRSV-N protein level, whereas the miR-novel-029 inhibitor did not significantly affect PRRSV-N protein levels (Figure 1D). Consistent with these results, the viral RNA levels were significantly elevated following treatment with the miR-191-5p inhibitor, unlike with the miR-novel-029 inhibitor (Figure 1E). These observations prompted us to focus our subsequent research on miR-191-5p. Additionally, we assessed the cytotoxic effects of the miRNA inhibitors and mimics and found that neither the inhibitors nor the mimics exhibited any cytotoxicity on PAMs (Figures 1C,F), ensuring the safety of these treatments for further study.

### 3.2 miR-191-5p suppresses PRRSV infection by targeting the replication phase

**Figure 2:**
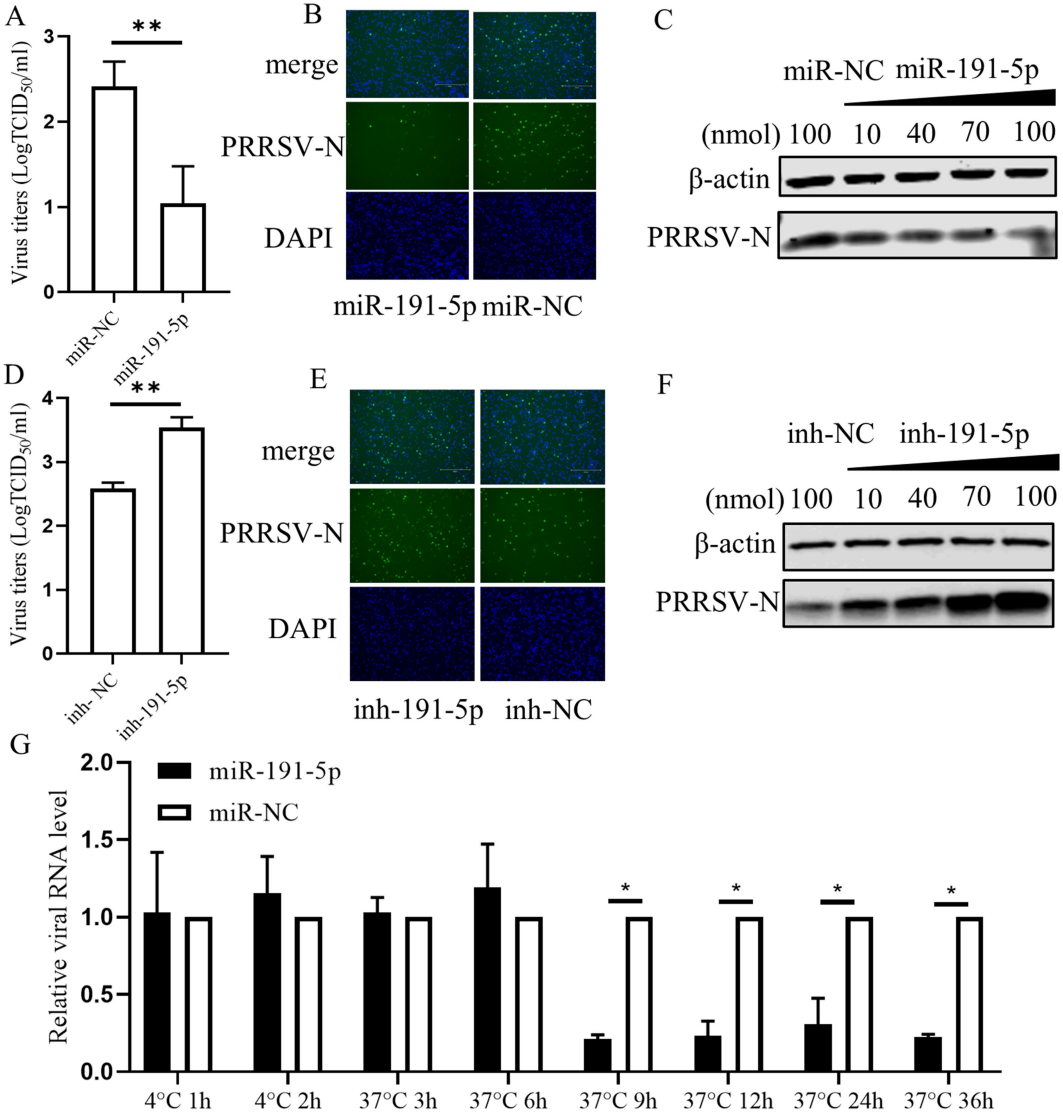
miR-191-5p Inhibits PRRSV replication. **(A,B)** miR-191-5p mimic represses PRRSV infection. PAMs were transfected with miR-191-5p mimic or miR-NC for 24 h, and the cell monolayers were then inoculated with HuN4 at MOI of 0.1 for 24 h. Cells supernatants were collected for detection of viral titers **(A)**. Virus infection was examined by IFA **(B)**. **(C)** miR-191-5p mimic dose-dependently inhibits PRRSV. PRRSV-N protein was detected by transfection with different miR-191-5p mimic concentrations. **(D,E)** miR-191-5p inhibitor promotes PRRSV infection. PAMs were transfected with miR-191-5p inhibitor or inhibitor-NC for 24 h, followed by inoculation with HuN4 at MOI of 0.1 for 24 h. Cells supernatants were collected for determined viral titer **(D)**. **(E)** Virus infection was examined by IFA. **(F)** miR-191-5p inhibitor promotes PRRSV. PRRSV-N protein was detected by transfection with different concentrations of miR-191-5p inhibitor. **(G)** Overexpression of miR-191-5p decreases PRRSV replication. Transfection of miR-191-5p was followed by inoculation of HuN4 with MOI of 0.1, and then cells were collected at different time points. The relative level of viral RNA was determined by qPCR. Data are presented as the mean ± SD. ^∗^*p* < 0.05; ^∗∗^*p* < 0.01.

Given the substantial reduction in PRRSV infection by the miR-191-5p mimic, we further investigate the regulatory function of miR-191-5p on PRRSV replication. Virus titers of supernatants from infected cells were measured by TCID_50_ assay, with confirmatory analysis on fixed cells via IFA. The TCID_50_ results showed that miR-191-5p treatment resulted in a pronounced decrease in PRRSV titers compared with miRNA negative control, with IFA results aligning with the TCID_50_ data (Figures 2A,B). The overexpression of miR-191-5p inhibited PRRSV infection in a dose-dependent manner (Figure 2C). Conversely, the inhibition of endogenous miR-191-5p significantly enhanced PRRSV infection compared to the negative control (Figures 2D,E). Similarly, the downregulation of miR-191-5p promoted PRRSV infection in a dose-dependent manner (Figure 2F). These outcomes demonstrated miR-191-5p’s antiviral efficacy against PRRSV. Further investigation of viral RNA levels at various intervals post-infection revealed that miR-191-5p’s inhibitory effect on PRRSV is detectable as early as at the 9 hpi, suggesting its interference with the later phases of the PRRSV replication cycle (Figure 2G). These findings indicate the potential of miR-191-5p as an inhibitor of PRRSV replication, particularly affecting viral replication in PAMs at later stages.

### 3.3 miR-191-5p targets porcine EGFR expression

**Figure 3:**
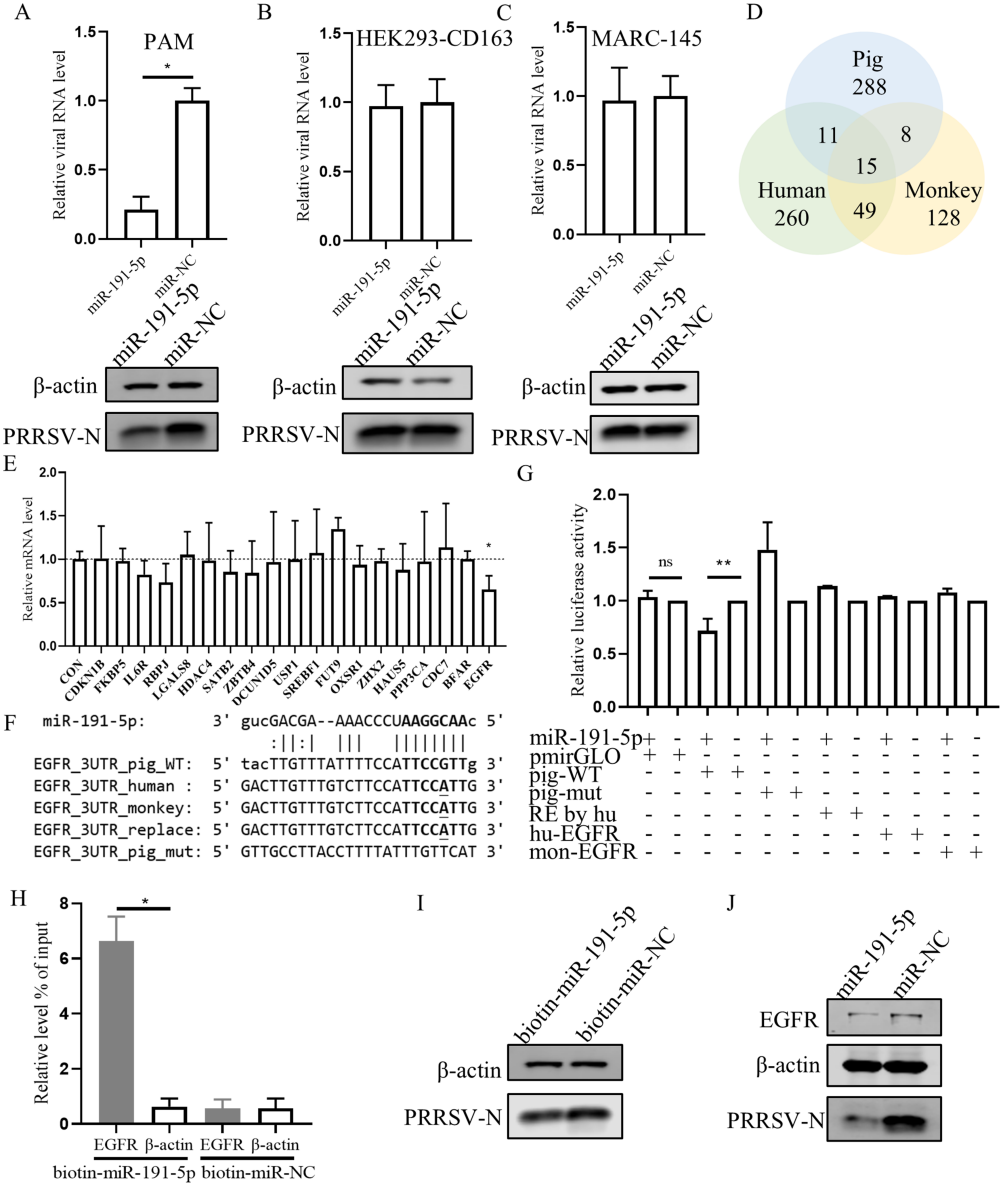
miR-191-5p negatively regulates the expression of porcine EGFR. **(A–C)** miR-191-5p mimic represses PRRSV infection in PAMs but not MARC-145 or HEK293-CD163. After transfection of miR-191-5p or miR-NC mimic into PAM **(A)**, HEK293-CD163 **(B)** and MARC-145 **(C)** for 24 h, and the cells were then infected with PRRSV at MOI of 0.1 for 24 h. Cells lysates were subjected to western blot to detect the expression of PRRSV-N protein. Relative viral RNA level was determined by qPCR. **(D)** Wayne plots of miR-191-5p 3’UTR predicted target in pig, human and monkey. **(E)** EGFR was downregulated by miR-191-5p. PAMs were transfected with miR-191-5p or miR-NC for 24 h. Total RNA was extracted, and the mRNA level were determined by qPCR. **(F)** diagram of the predicted miR-191-5p binding sites in EGFR 3’UTR of different species. miR-191-5p seed region and its predicted binding sites are indicated in bold. Bases with mutations and differences in seed prediction binding regions are underlined. **(G)** miR-191-5p directly targets EGFR 3’UTR. HEK293T cells were co-transfected with EGFR 3’UTR wild type or mutant luciferase reporter vector (100 ng), along with miR-191-5p mimics or NC for 48 h and then harvested for luciferase assay. **(H)** miR-191-5p binds to 3’UTR of EGFR. PAMs were transfected with biotin-labeled miR-191-5p or control (biotin-miR-NC). After 36 h the cells were collected for miRNA-pulldown assay and EGFR and *β*-actin mRNA level were detected using qPCR. **(I)** Inhibition of PRRSV by biotin-miR-191-5p. After transfection of biotin-miR-191-5p into PAMs, the PRRSV-N protein was detected by western blot. **(J)** miR-191-5p decreases EGFR protein. After miR-191-5p transfection for 24 h, the cell monolayers were then inoculated with HuN4 at MOI of 0.1 for 24 h. Cell lysates were subjected to western blot to detect PRRSV-N and EGFR protein level. Data are presented as the mean ± SD. ns, *p* > 0.05; ^∗^*p* < 0.05; ^∗∗^*p* < 0.01.

Previous studies have proved that numerous mammalian mRNAs are conserved targets for miRNAs regulation. Here, miR-191-5p mimic was transfected into PAMs (porcine cell), MARC-145 (monkey cell), and HEK293-CD163 (human cell). The results showed that PRRSV replication was inhibited by the miR-191-5p mimic specifically in PAMs, but not in MARC-145 or HEK293-CD163 (Figures 3A–C). This suggests that the mRNA targets of miR-191-5p may not be conserved across pig, monkey, and human. Then we used miRanda (Enright et al., 2003) to predicted potential miR-191-5p targets in three pieces (Supplementary Table S1), and obtained 288 specific mRNA candidates in pigs (Figure 3D). Further, we collect literature on the predicted gene related to viral or immune functions by GeneRIF (Supplementary Tables S2, S3; Mitchell et al., 2003). The qPCR analysis showed that the expression of EGFR was decreased upon transfection with miR-191-5p (Figure 3E). We compared the predicted the binding site of miR-191-5p on pig EGFR-3’UTR with those of human and monkey, and found that the pig sequence was different from the human and monkey as expected (Figure 3F). Dual-luciferase assays, a common method to assess miRNAs-target gene interactions, were constructed using a dual-luciferase reporter vector pmirGLO containing 3’UTR sequence of EGFR mRNA (Supplementary Table S4). HEK293T were co-transfected with either an empty vector or a wild-type EGFR mRNA 3’UTR plasmid in the presence of miR-191-5p mimic or miRNA negative control (miR-NC) for 48 h. The results displayed that miR-191-5p mimic significantly downregulated the luciferase activity of the pig EGFR wide-type 3’UTR relative to the control, but failed to affect the EGFR 3’UTR of human and monkey. The inhibition effect of miR-191-5p mimic on the luciferase activity was abolished by pig mutant (Figure 3G). The mRNA level of EGFR was significantly higher in the complex precipitated with biotinylated miR-191-5p than with the biotinylated miRNA negative control, while the mRNA level of *β*-actin remained unchanged (Figure 3H). Correspondingly, biotinylated miR-191-5p significantly inhibited PRRSV at the protein level (Figure 3I). We also found miR-191-5p significantly decreased both EGFR and PRRSV protein levels (Figure 3J). Taken together, these results demonstrate that miR-191-5p inhibits PRRSV replication specifically in pig cells, suggesting that EGFR might be a target of miR-191-5p in the antivirus process.

### 3.4 Inhibition of EGFR negatively regulates PRRSV replication

**Figure 4:**
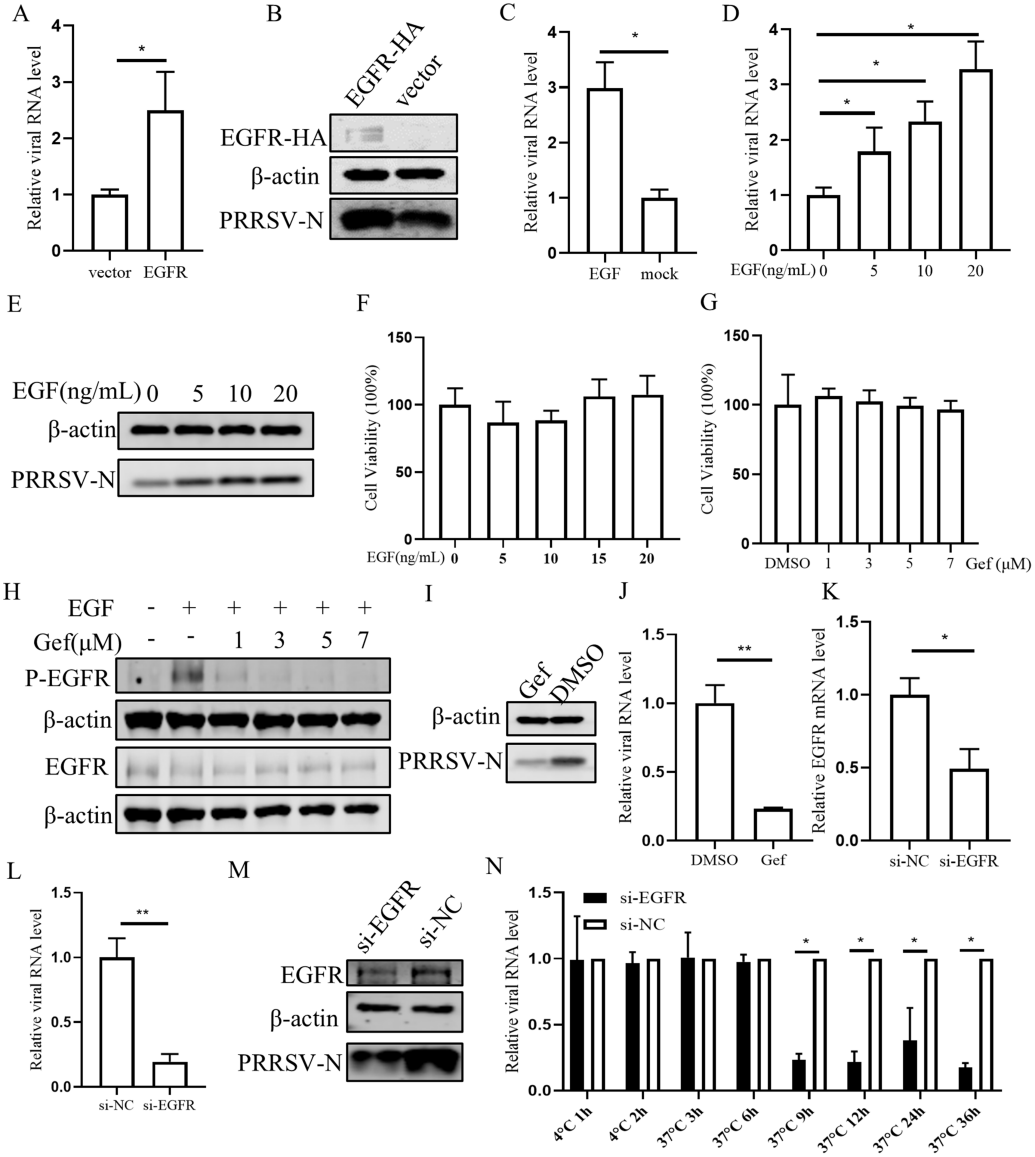
Inhibition of Endogenous EGFR suppresses PRRSV replication. **(A,B)** EGFR promotes PRRSV infection. PAMs were incubated with EGFR lentivirus mixed with polybrene for 12 h and then infected with PRRSV for 24 h. Cells were collected to detect PRRSV RNA and protein separately. **(C–E)** EGFR facilitates PRRSV infection. PAMs were stimulated with different concentrations of EGF and then inoculated with PRRSV for 24 h. Cells were collected to detect PRRSV RNA and protein separately. **(F,G)** EGF and gefitinib have no effect on PAMs’s viability. CCK8 assay was performed on PAMs treated with EGF or gefitinib for 72 h at indicated concentration. **(H)** Inhibition of EGFR phosphorylation by gefitinib in PAMs. After 24 h of gefitinib pretreatment, PAMs were stimulated with EGF for 30 min and then harvested for western blot detection of protein expression. **(I,J)** Gefitinib inhibits PRRSV infection. PAMs were treated with gefitinib at a concentration of 7 μM for 24 h, the cells were then inoculated with PRRSV for another 24 h. The cells were collected to detect PRRSV-N protein **(I)** and RNA **(J)**. **(K)** Verification of EGFR knocking down. PAMs were transfected with EGFR-specific siRNA (si-EGFR) or si-NC for 24 h, and the knockdown efficiency of EGFR was determined by qPCR. **(L,M)** Knocking down EGFR decreases PRRSV infection. After transfection of si-EGFR for 24 h, PAMs were inoculated with PRRSV for 24 h. The cells were collected to detect PRRSV RNA **(L)** and protein level **(M)**. **(N)** Depletion of endogenous EGFR decreases PRRSV replication. After si-EGFR knockdown of EGFR, PAMs were collected at different time points following PRRSV inoculation. Data are presented as the mean ± SD. ^∗^*p* < 0.05; ^∗∗^*p* < 0.01.

Next the role of EGFR in PRRSV infection was investigated. Initially, PAMs were transfected with EGFR using a lentivirus vector. The results confirmed the successful overexpression of EGFR, which resulted in an increase in both PRRSV RNA and protein levels in PAMs (Figures 4A,B). As EGFR is a tyrosine kinase receptor that can be activated by EGF, we stimulated PAMs with EGF and subsequently inoculated them with PRRSV. This treatment led to an increase in viral RNA, indicating that EGFR activation can enhance PRRSV replication (Figure 4C). Further, we investigated the effect of different EGF concentrations and found that EGF dose-dependently promoted PRRSV replication (Figures 4D,E). The cytotoxic of EGF was assessed and no significant toxicity of EGF was evident at concentrations of <20 ng/mL (Figure 4F). To further determine the effect of EGFR on PRRSV infection, we next exposed PAMs to the EGFR inhibitor gefitinib (Bean et al., 2007). The cell cytotoxicity result showed that gefitinib, at concentration below 7 μM did not affect the viability of PAMs (Figure 4G). PAMs were then treated with different concentrations of gefitinib and followed by PRRSV infection. The results showed that gefitinib dose-dependently inhibited EGFR phosphorylation (Figure 4H) and the gefitinib significantly decreased the levels of viral RNA and protein (Figures 4I,J). To directly address the role of EGFR, we designed siRNA to knockdown EGFR expression. The qPCR result showed that si-EGFR treatment significantly decreased EGFR levels (Figure 4K). After infection with PRRSV, we observed that viral infection was decreased in EGFR-knockdown PAMs compared to those treated with a non-targeting siRNA (si-NC) (Figures 4L,M). To further examine the effect of EGFR on PRRSV infection, we examined the viral RNA levels at different time points post-infection in EGFR-knockdown PAMs. The viral RNA levels were significantly reduced after 9 hpi (Figure 4N), suggesting that EGFR-knockdown inhibition affects the stage of viral biosynthesis. Together, these results are consistent with the inhibitory effects observed with miR-191-5p and demonstrate that inhibition of EGFR negatively regulates PRRSV replication.

### 3.5 miR-191-5p abrogates the inhibitory effect of EGFR on IFN-*β*, consequently inhibiting PRRSV replication

**Figure 5:**
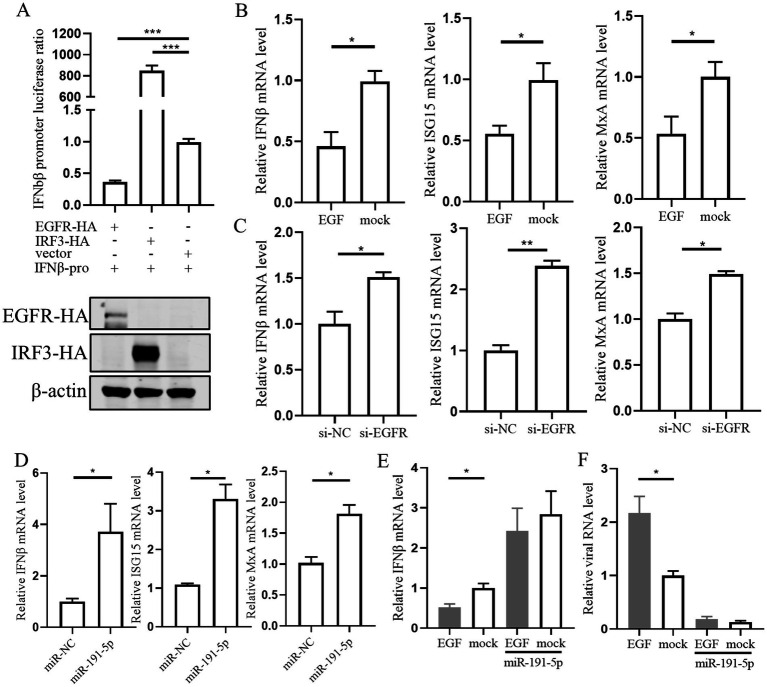
miR-191-5p regulates viral replication by modulating interferon activity through EGFR. **(A)** EGFR inhibit IFN-β promoter luciferase activity. The IFN-β promoter and pRL-TK were co-transfected into HEK293T cells along with EGFR, IRF3, and control, respectively. The cells were collected for luciferase activity assay after 48 h. **(B)** PAMs were inoculated with 20 ng/mL of EGF for 24 h, and the cells were collected for qPCR to detect the RNA level of the indicated gene. **(C,D)** PAMs were transfected with miR-191-5p or si-EGFR for 24 h. The RNA level was determined by qPCR. **(E)** PAMs were transfected with miR-191-5p and after 24 h the cells were treated with EGF. Total RNA was extracted, and the mRNA levels of IFN-β, were determined by qPCR. **(F)** PAMs were treated with EGF, then cells were infected with PRRSV for 24 h. Total RNA was extracted to detect the expression levels of PRRSV RNA. Data are presented as the mean ± SD. ^∗^*p* < 0.05; ^∗∗^*p* < 0.01; ^∗∗∗^*p* < 0.001.

To understand the regulatory role of EGFR in antiviral response, IFN-β promoter activities were evaluated. The results showed that IFN-β promoter was robustly activated by the transcription factor IRF3, yet it was significantly inhibited by the EGFR (Figure 5A). We then examined the mRNA level of IFN-β, myxovirus resistance protein A (MxA), and the interferon-stimulated gene 15 (ISG15) in PAMs stimulated with EGF or transfected with miR-191-5p or si-EGFR. The qPCR results showed a significant reduction in the mRNA levels of IFN-*β*, MxA, and ISG15 in EGF-treated PAMs (Figure 5B). In contrast, transfection with miR-191-5p or si-EGFR significantly increased the mRNA levels of these genes (Figures 5C,D). To further explore the role of miR-191-5p in modulating the effects of EGFR on viral infection and IFN-*β* expression, we overexpressed miR-191-5p before exposing PAMs to EGF stimulation. Our results demonstrate that miR-191-5p effectively attenuated the inhibitory effect of EGF on interferon signaling and inhibited the promotional effect of EGF on PRRSV replication (Figures 5E,F). These results suggest that miR-191-5p plays a pivotal role in modulating the inhibitory effects of EGFR on IFN-I, thereby inhibiting PRRSV replication.

### 3.6 Inhibition of STAT3 suppresses PRRSV infection

**Figure 6:**
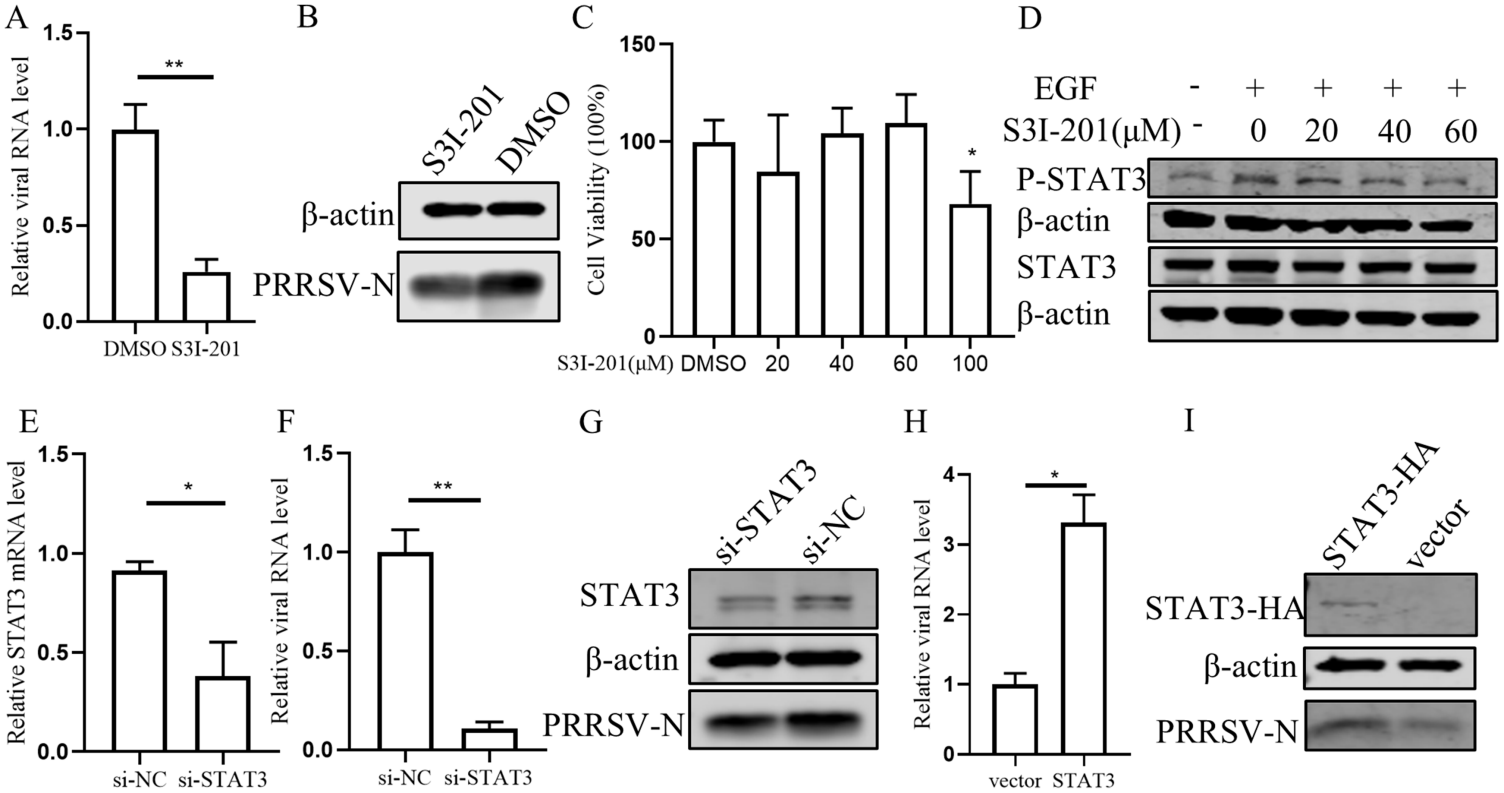
STAT3 promotes PRRSV replication in PAMs. **(A,B)** The effect of STAT3 inhibitor on PRRSV. PAMs were pretreated with 60 μM S3I-201 for 24 h and then inoculated with PRRSV at 0.1 MOI for 24 h. Cells were collected to detect the expression levels of PRRSV RNA **(A)** and protein **(B)**. **(C)** The effect of S3I-201 on cell viability. CCK8 assay was performed on PAMs treated with S3I-201 for 72 h at the indicated concentration. **(D)** PAMs were pretreatment with S3I-201 for 24 h and stimulated with EGF for 30 min. Cells were then harvested for western blot to detect the indicated protein expression. **(E)** Verification of si-STAT3 knockdown efficiency. The mRNA level of STAT3 was detected in PAMs after transfection of si-STAT3 for 24 h. **(F,G)** Effect of knocking down endogenous STAT3 on PRRSV. PAMs were transfected with si-STAT3 for 24 h and then infected with PRRSV for 24 h. Cells were collected to detect the expression levels of PRRSV RNA **(F)** and protein **(G)**. **(H,I)** STAT3 promotes PRRSV infection. PAMs were incubated with STAT3 lentivirus mixed with polybrene for 12 h and then infected with PRRSV for 24 h. Cells were collected to detect PRRSV RNA **(H)** and protein **(I)** separately. Data are presented as the mean ± SD. ^∗^*p* < 0.05; ^∗∗^*p* < 0.01.

According to previous research that EGFR can modulate the antiviral response of IFN-I through regulating STAT3 (Yang et al., 2018), we conducted a series of experiments to investigate the role of STAT3 in PRRSV replication in PAMs. Initially, PAMs were treated with STAT3 inhibitor S3I-201 and subsequently infected with PRRSV. We observed that the levels of viral RNA and protein were significantly decreased in the presence of the S3I-201 as compared those treated with the control DMSO, indicating that STAT3 inhibition impairs PRRSV replication (Figures 6A,B). Further, we observed that S3I-201, at concentrations below 60 μM, did not adversely affect cell’s viability (Figure 6C), and that the STAT3 inhibitor S3I-201 dose-dependently inhibited STAT3 phosphorylation (Figure 6D). To substantiate these findings, we designed siRNA to knockdown STAT3, which resulted in a significant decrease in STAT3 mRNA levels and a corresponding reduction in PRRSV RNA and protein levels (Figures 6E–G). Conversely, overexpression of STAT3 led to an increase in PRRSV RNA and protein levels, confirming that STAT3 promotes PRRSV replication (Figures 6H,I). These results indicate that STAT3 has the capability to enhance PRRSV replication.

### 3.7 STAT3 is a convergent node in the miR-191-5p-EGFR axis in regulating interferon signaling and PRRSV infection

**Figure 7:**
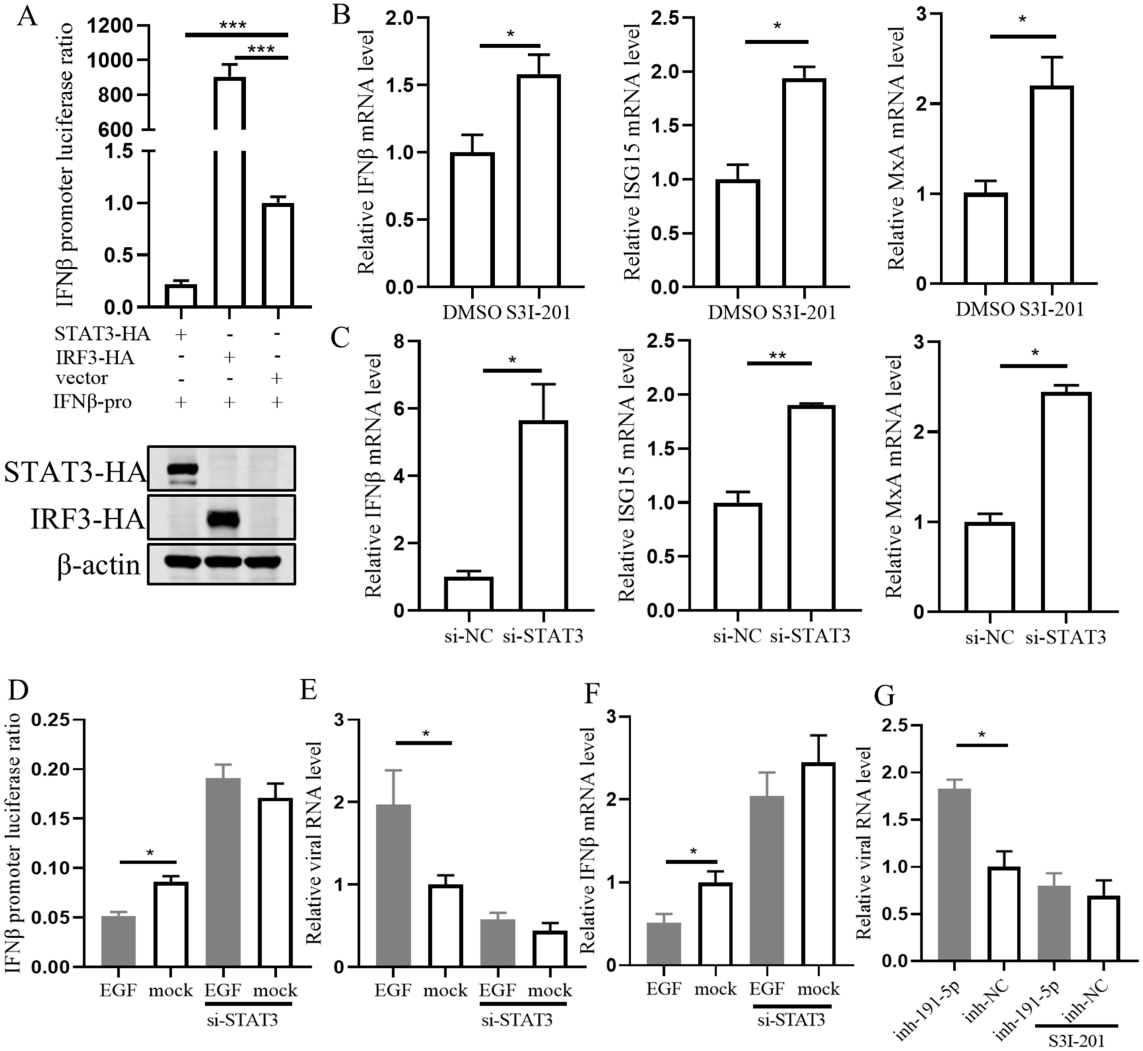
STAT3 is downstream of EGFR in regulating interferon signaling during EGF activation in PAMs. **(A)** STAT3 inhibits IFN-β promoter luciferase activity. The IFN-β promoter and pRL-TK were co-transfected into HEK293T along with STAT3, IRF3, and control, respectively. The cells were collected for luciferase activity assay after 48 h. **(B,C)** PAMs were treated with the inhibitor S3I-201 or transfected with si-STAT3 for 24 h. RNA was extracted to detect the expression levels of indicated mRNA using qPCR. **(D)** Immortalized PAM were co-transfected with the IFN-β promoter and pRL-TK along with si-STAT3 or si-NC for 12 h, and then treated with EGF or control for another 24 h. The cells were collected for luciferase activity assay. **(E)** PAMs were transfected with si-STAT3 for 12 h, followed by treatment with EGF for 24 h, and the cells were collected for detection of IFN-β mRNA level. **(F)** PAMs were treated with EGF, then cells were infected with PRRSV for 24 h. Total RNA was extracted to detect the expression levels of PRRSV RNA. **(G)** PAMs were treated with S3I-201 for 12 h, then transfected with miR-191-5p inhibitor or NC for 12 h, followed by infection with PRRSV for 24 h. The cells were collected for detection of PRRSV RNA level. Data are presented as the mean ± SD. ^∗^*p* < 0.05; ^∗∗^*p* < 0.01; ^∗∗∗^*p* < 0.001.

Previous studies have reported that STAT3 can inhibit interferon signaling in several cell types (Lupberger et al., 2013; Yang et al., 2018). Therefore, we assessed the impact of STAT3 overexpression on the luciferase activity of the pig IFN-*β* promoter and observed a significant inhibition (Figure 7A). To clarify whether the effect of STAT3 was mediated through the IFN-I signaling pathway in PAMs, we treated cells with the STAT3 inhibitor S3I-201 or transfected them with si-STAT3. The result showed that the levels of IFN-β, ISG15, and MxA were significantly increased (Figures 7B,C), suggesting that STAT3’s inhibitory effect is indeed dependent on the impairment of IFN-I signaling. Further investigation into STAT3’s role in IFN-β regulation downstream of EGFR revealed that knocking down STAT3 attenuated the inhibitory effect of EGF on IFN-β and its enhancement of PRRSV replication (Figures 7D–F). This suggests that STAT3 functions downstream of EGFR in the regulation of IFN-β expression. Additionally, S3I-201 (STAT3 inhibitor) significantly reduced the promotional effect of miR-191-5p inhibitor on PRRSV (Figure 7G), indicating that miR-191-5p may regulate IFN-β through the EGFR-STAT3 pathway, with STAT3 being an integral component of this regulatory mechanism.

### 3.8 miR-191-5p broadly inhibits different PRRSV strains

**Figure 8:**
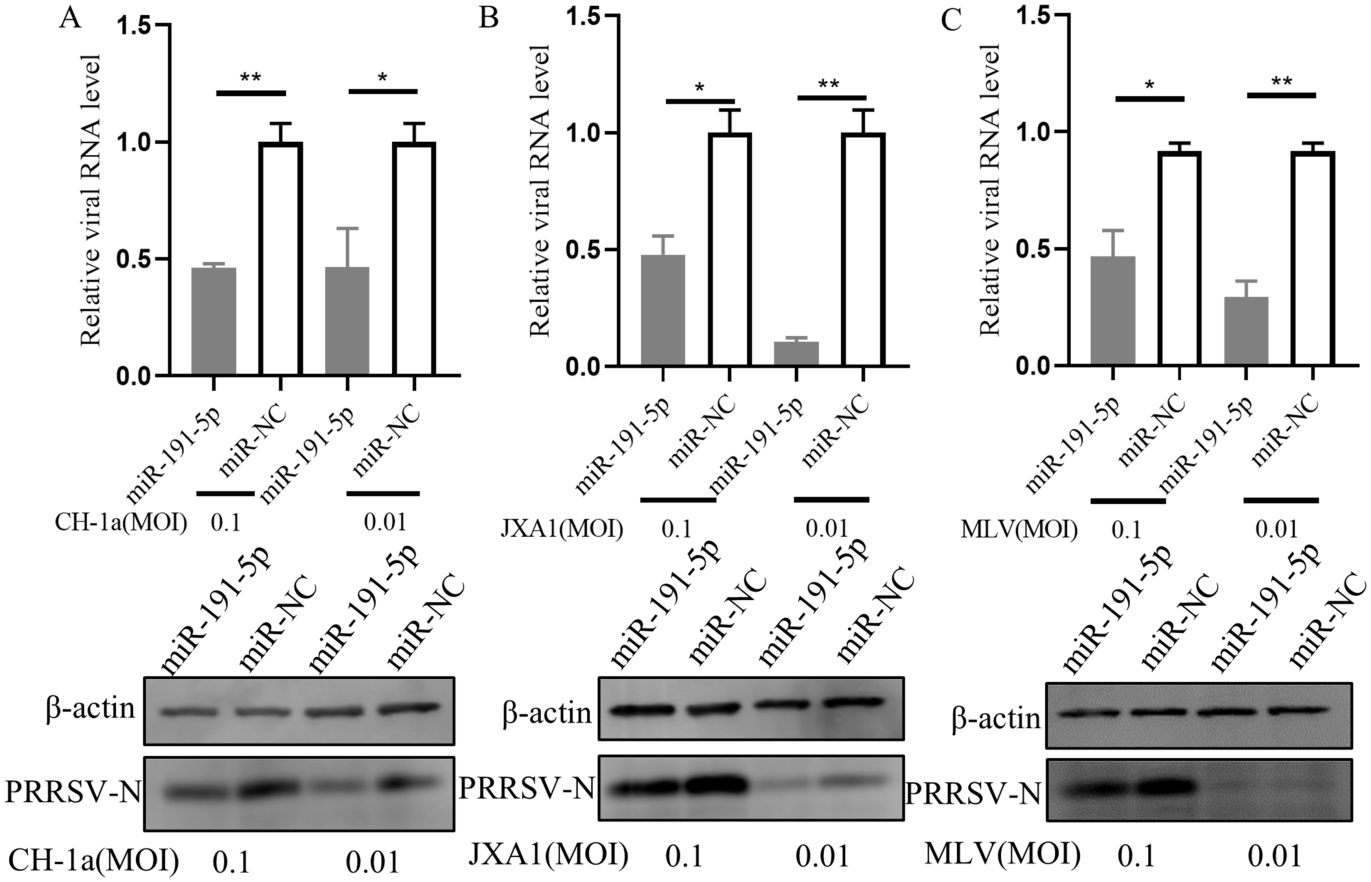
miR-191-5p broadly inhibits various PRRSV strains. **(A–C)** miR-191-5p overexpression decreases CH-1a, JXA1, and MLV replication. PAMs were transfected with miR-191-5p or miR-NC for 24 h and then infected with the indicated PRRSV strains at 0.1 or 0.01 MOI for another 24 h. Cells were collected, and PRRSV RNA and protein were detected separately. Data are presented as the mean ± SD. ^∗^*p* < 0.05; ^∗∗^*p* < 0.01.

Next, we explored the capacity of miR-191-5p to exert a broad-spectrum inhibitory effect against multiple PRRSV strains. PAMs were first transfected with miR-191-5p for a period of 24 h. Following transfection, the cells were inoculated with three distinct PRRSV strains: CH-1a (traditional Chinese strain), JXA1 (another HP-PRRSV strain) and MLV (North American vaccine strain), each at an MOI of 0.1 or 0.01, and the cultures were incubated for another 24 h. The results showed that miR-191-5p effectively inhibited the replication of all three PRRSV strains in PAMs (Figures 8A–C). These results indicate that miR-191-5p possesses the potential to broadly inhibit a variety of PRRSV strains, highlighting its therapeutic potential in combating this viral infection.

## 4 Discussion

Current studies have shown that miRNAs are involved in a variety of physiological and pathological processes (Lei et al., 2022; Zhang J. et al., 2023). Moreover, miRNAs play a crucial role in regulating host-virus interactions (Mishra et al., 2019; Barbu et al., 2020; Bauer et al., 2023). Certain miRNAs such as miR-181, miR-23, miR-378, and miR-505 inhibit viral sequences directly (Guo et al., 2013; Zhang et al., 2014). Other miRNAs, including miR-218, microRNA-30c, and miR-140, exhibit antiviral activity by regulating mRNA sequences of key proteins involved in viral infection or are linked to the immune system (Zhang et al., 2016; Xu et al., 2020; Zhang et al., 2021). However, there are still many other pig miRNAs remain to be fully elucidated. In this study, we identified miR-191-5p from our laboratory’s miRNA library, which inhibits PRRSV replication.

During viral infection, miRNAs are deeply involved in various stages of the viral life cycle as important intracellular components. In 2003, researchers first reported miR-191-5p in mice, and since then, it has been detected in several other species (Lagos-Quintana et al., 2003; Kiezun et al., 2012). Subsequent research has revealed that miR-191-5p is involved in numerous biological processes, including growth, development, and cancer progression (Nagpal and Kulshreshtha, 2014). For example, miR-191-5p regulates cortical development by targeting brain-derived neurotrophic (factor BDNF) (Mellios et al., 2008), and promotes the proliferation and migration of human breast cancer cells by targeting special AT-rich sequence-binding protein-1 (SATB1) (Nagpal et al., 2013). Additionally, miR-191-5p has been identified as a marker for various tumors (Taguchi and Murakami, 2013; Majed, 2022). In mammals, target sequences of many miRNAs identified previously are typically conserved (Zhang et al., 2016; Zhang et al., 2021). Our results revealed that miR-191-5p specifically targeted the 3’UTR of EGFR mRNA of porcine, while having no effect on the corresponding sites in humans and monkeys. This non-conservative targeting effect is not unique; it has been observed that the 3′UTR of EGFR is poorly conserved among human, mice, and rat, with microRNA-7 targeting only the human EGFR mRNA 3′UTR (Webster et al., 2009). Large scale biochemical characterization of mammalian microRNA targets has shown that approximately 7% of target sites are non-canonical (Chi et al., 2009; Hafner et al., 2010). The researchers utilized mirSVR to further support the notion that non-canonical sites account for a significant portion of microRNA-mediated silencing (Betel et al., 2010). This finding indicates that miR-191-5p is not conserved in mammalian EGFR. Previous studies have only shown that miR-191-5p inhibits HIV by targeting nucleoporin 50 (Nup50) (Zheng et al., 2021). However, miR-191-5p has not been studied in the context of other viruses. Furthermore, Nup50 is an important component of the nuclear pore complex, and miR-191-5 inhibits viral replication by down-regulating NUP50 to inhibit HIV entry into the nucleus which is independent of IFN-I signaling (Dickson et al., 2024). Interestingly, our results revealed that miR-191-5p regulates IFN-*β* expression and inhibits PRRSV replication.

Given that miR-191-5p can directly bind to porcine EGFR mRNA, we explored the role of EGFR in the host, particularly its function during viral infection. The previous reports showed that EGFR plays a critical role in pathological processes through its various downstream signaling pathways. For instance, mutations in EGFR lead to prolonged activation of the PI3K/Akt/mTOR pathway, which promotes Glioblastoma multiform (GBM) development and drug resistance (Li et al., 2016). Additionally, activation of the EGFR-RAS–RAF–MEK-MAPK cascade promotes cancer cell proliferation, survival, migration and angiogenesis through the transcriptional regulation of related genes (Martinelli et al., 2017). In our experiments, we observed that knockdown of EGFR in PAMs not only inhibited PRRSV replication but also promoted interferon expression. This finding is consistent with studies on HCV and JEV, where EGFR was shown to inhibit the interferon response and promote viral replication (Lupberger et al., 2013; Zhang et al., 2022). Although, previous studies indicated that EGFR enhances PRRSV invasion in MARC-145 (Wang et al., 2016). However, our results demonstrated that EGFR knockdown primarily affected the replication phase of the virus in PAMs. Macrophages not only serve as an important barrier for natural immunity, but also play an important role in the regulation of the interferon signaling. EGFR is highly expressed mainly in epithelial cells, with lower expression in macrophages. We therefore hypothesized that EGFR protein expression in PAMs is much lower than that in MARC-145 cells, leading to inconsistency between our experimental results and the inhibition of viral invasion by EGFR knockdown observed in MARC-145 cells. Zhang et al. found that in immortalized PAMs, inhibition of JEV by knockdown of EGFR occurred at 8–24 h of viral infection, in agreement with our finding that EGFR can regulate viral replication via interferon (Zhang et al., 2022). Further, we combined EGF with miR-191-5p and found that the effect of activated EGFR on IFN-*β* and virus was inhibited by miR-191-5p.

The main downstream pathways of EGFR activation include phosphatidylinositol 3-kinase (PI3K)-Akt–mTOR, Ras–Raf–MEK, and JAK2-STAT3 (Martinelli et al., 2017; Jensen et al., 2020; Bang et al., 2023; Bi et al., 2023). STAT3, a downstream of EGFR, has been involved in PRRSV-host interaction. Furthermore, previous studies have shown that the NSP5 protein of PRRSV ubiquitinates STAT3 for degradation (Yang et al., 2017), but there is no clear evidence that STAT3 regulates PPRSV infection. Our findings revealed that PRRSV replication can be inhibited by either blocking STAT3 phosphorylation or knocking down of STAT3. A similar phenomenon is observed with other viruses. Blocking STAT3 phosphorylation inhibited varicella-zoster virus replication and spread, and knocking down STAT3 inhibited PEDV replication (Sen et al., 2012; Yang et al., 2018). They noted that reduced STAT3 phosphorylation associated with enhanced expression of SOCS3, Mx1, and radical S-adenosyl methionine domain containing 2 (RSAD2), suggesting that inhibition of EGFR signaling affects STAT3 phosphorylation, leading to enhanced expression of ISGs and antiviral activity, which is consistent with our findings. However, current research on downstream molecules of EGFR-regulated IFN-β signaling reveals a variety of perspectives (Gong et al., 2020; Zhang et al., 2022; Pan et al., 2024). We focused on the role of STAT3 in EGFR-regulated interferon expression. Whether other downstream molecules of EGFR are involved in this process requires further investigation. Finally, the effect of miR-191-5p inhibitor on PRRSV was diminished when STAT3 phosphorylation was inhibited by S3I-201. This finding confirms that miR-191-5p regulates IFN-β expression and PRRSV replication via the EGFR-STAT3 pathway, though other pathways may also be involved in the regulation of IFN-β expression and PRRSV infection.

However, there are several limitations of the present study. IFN-β plays a central role in natural immune antagonism against viruses, and receives multiple signals to modulate the immune response through different downstream genes. Further analysis of miR-191-5p predicted target genes revealed that miR-191-5p predicted target genes are enriched in the natural immune pathway, and thus we speculate that there are more miR-191-5p targets involved in this process. The study of the precise and enriched regulatory network of miR-191-5p and its antagonistic viral mechanism will be the direction of our further research.

In summary, our experiments demonstrate that miR-191-5p plays a significant role in the host response to PRRSV infection. Specifically, it down-regulates the expression of porcine EGFR and up-regulates interferon, thereby inhibiting PRRSV replication. This study not only highlight the crucial role of miR-191-5p in regulating IFN-I signaling pathway but also enriches the theoretical foundation for understanding miRNA’s involvement in host-virus interactions, presenting miR-191-5p as a promising therapeutic target for PRRSV.

## Data Availability

The original contributions presented in the study are included in the article/Supplementary material, further inquiries can be directed to the corresponding authors.

## References

[ref1] Bang J. Jun M. Lee S. Moon H. Ro S. W. (2023). Targeting EGFR/PI3K/AKT/mTOR signaling in hepatocellular carcinoma. Pharmaceutics 15:2130. doi: 10.3390/pharmaceutics15082130, PMID: 37631344 PMC10458925

[ref2] Barbu M. G. Condrat C. E. Thompson D. C. Bugnar O. L. Cretoiu D. Toader O. D.. (2020). MicroRNA involvement in signaling pathways during viral infection. Front. Cell Dev. Biol. 8:143. doi: 10.3389/fcell.2020.00143, PMID: 32211411 PMC7075948

[ref3] Bauer A. N. Majumdar N. Williams F. Rajput S. Pokhrel L. R. Cook P. P.. (2023). MicroRNAs: small but key players in viral infections and immune responses to viral pathogens. Biology 12:1334. doi: 10.3390/biology12101334, PMID: 37887044 PMC10604607

[ref4] Bean J. Brennan C. Shih J. Y. Riely G. Viale A. Wang L.. (2007). MET amplification occurs with or without T790M mutations in EGFR mutant lung tumors with acquired resistance to gefitinib or erlotinib. Proc. Natl. Acad. Sci. U. S. A. 104, 20932–20937. doi: 10.1073/pnas.0710370104, PMID: 18093943 PMC2409244

[ref5] Betel D. Koppal A. Agius P. Sander C. Leslie C. (2010). Comprehensive modeling of microRNA targets predicts functional non-conserved and non-canonical sites. Genome Biol. 11:R90. doi: 10.1186/gb-2010-11-8-r90, PMID: 20799968 PMC2945792

[ref6] Bi J. Wu Z. Zhang X. Zeng T. Dai W. Qiu N.. (2023). TMEM25 inhibits monomeric EGFR-mediated STAT3 activation in basal state to suppress triple-negative breast cancer progression. Nat. Commun. 14:2342. doi: 10.1038/s41467-023-38115-2, PMID: 37095176 PMC10126118

[ref7] Cai H. Zhang H. Cheng H. Liu M. Wen S. Ren J. (2023). Progress in PRRSV infection and adaptive immune response mechanisms. Viruses 15:1442. doi: 10.3390/v15071442, PMID: 37515130 PMC10385784

[ref8] Chi S. W. Zang J. B. Mele A. Darnell R. B. (2009). Argonaute HITS-CLIP decodes microRNA-mRNA interaction maps. Nature 460, 479–486. doi: 10.1038/nature08170, PMID: 19536157 PMC2733940

[ref9] Corzo C. A. Mondaca E. Wayne S. Torremorell M. Dee S. Davies P.. (2010). Control and elimination of porcine reproductive and respiratory syndrome virus. Virus Res. 154, 185–192. doi: 10.1016/j.virusres.2010.08.016 20837071

[ref10] Dash S. Balasubramaniam M. Dash C. Pandhare J. (2018). Biotin-based pulldown assay to validate mRNA targets of cellular miRNAs. J. Vis. Exp. 136:57786. doi: 10.3791/57786, PMID: 29985341 PMC6101703

[ref11] Dickson C. F. Hertel S. Tuckwell A. J. Li N. Ruan J. Al-Izzi S. C.. (2024). The HIV capsid mimics karyopherin engagement of FG-nucleoporins. Nature 626, 836–842. doi: 10.1038/s41586-023-06969-7, PMID: 38267582 PMC10881392

[ref12] Enright A. J. John B. Gaul U. Tuschl T. Sander C. Marks D. S. (2003). MicroRNA targets in Drosophila. Genome Biol. 5:R1. doi: 10.1186/gb-2003-5-1-r1, PMID: 14709173 PMC395733

[ref13] Eulalio A. Huntzinger E. Izaurralde E. (2008). Getting to the root of miRNA-mediated gene silencing. Cell 132, 9–14. doi: 10.1016/j.cell.2007.12.024 18191211

[ref14] Gao X. You X. Wang G. Liu M. Ye L. Meng Y.. (2024). MiR-320 inhibits PRRSV replication by targeting PRRSV ORF6 and porcine CEBPB. Vet. Res. 55:61. doi: 10.1186/s13567-024-01309-7, PMID: 38750508 PMC11097481

[ref15] Gong K. Guo G. Panchani N. Bender M. E. Gerber D. E. Minna J. D.. (2020). EGFR inhibition triggers an adaptive response by co-opting antiviral signaling pathways in lung cancer. Nat. Cancer 1, 394–409. doi: 10.1038/s43018-020-0048-0, PMID: 33269343 PMC7706867

[ref16] Gregory R. I. Chendrimada T. P. Cooch N. Shiekhattar R. (2005). Human RISC couples microRNA biogenesis and posttranscriptional gene silencing. Cell 123, 631–640. doi: 10.1016/j.cell.2005.10.022, PMID: 16271387

[ref17] Guo X. K. Zhang Q. Gao L. Li N. Chen X. X. Feng W. H. (2013). Increasing expression of microRNA 181 inhibits porcine reproductive and respiratory syndrome virus replication and has implications for controlling virus infection. J. Virol. 87, 1159–1171. doi: 10.1128/jvi.02386-12, PMID: 23152505 PMC3554091

[ref18] Hafner M. Landthaler M. Burger L. Khorshid M. Hausser J. Berninger P.. (2010). Transcriptome-wide identification of RNA-binding protein and microRNA target sites by PAR-CLIP. Cell 141, 129–141. doi: 10.1016/j.cell.2010.03.009, PMID: 20371350 PMC2861495

[ref19] Janssen H. L. Reesink H. W. Lawitz E. J. Zeuzem S. Rodriguez-Torres M. Patel K.. (2013). Treatment of HCV infection by targeting microRNA. N. Engl. J. Med. 368, 1685–1694. doi: 10.1056/NEJMoa1209026 23534542

[ref20] Jensen K. V. Hao X. Aman A. Luchman H. A. Weiss S. (2020). EGFR blockade in GBM brain tumor stem cells synergizes with JAK2/STAT3 pathway inhibition to abrogate compensatory mechanisms in vitro and in vivo. Neurooncol. Adv. 2:vdaa020. doi: 10.1093/noajnl/vdaa020, PMID: 32226941 PMC7086303

[ref21] Katopodis P. Randeva H. S. Spandidos D. A. Saravi S. Kyrou I. Karteris E. (2022). Host cell entry mediators implicated in the cellular tropism of SARS-CoV-2, the pathophysiology of COVID-19 and the identification of microRNAs that can modulate the expression of these mediators (review). Int. J. Mol. Med. 49:20. doi: 10.3892/ijmm.2021.5075, PMID: 34935057 PMC8722767

[ref22] Kiezun A. Artzi S. Modai S. Volk N. Isakov O. Shomron N. (2012). miRviewer: a multispecies microRNA homologous viewer. BMC. Res. Notes 5:92. doi: 10.1186/1756-0500-5-92, PMID: 22330228 PMC3292992

[ref23] Lagos-Quintana M. Rauhut R. Meyer J. Borkhardt A. Tuschl T. (2003). New microRNAs from mouse and human. RNA 9, 175–179. doi: 10.1261/rna.2146903, PMID: 12554859 PMC1370382

[ref24] Lei L. Cheng A. Wang M. Jia R. (2022). The influence of host miRNA binding to RNA within RNA viruses on virus multiplication. Front. Cell. Infect. Microbiol. 12:802149. doi: 10.3389/fcimb.2022.802149, PMID: 35531344 PMC9069554

[ref25] Li Y. Fan X. He X. Sun H. Zou Z. Yuan H.. (2012). MicroRNA-466l inhibits antiviral innate immune response by targeting interferon-alpha. Cell. Mol. Immunol. 9, 497–502. doi: 10.1038/cmi.2012.35, PMID: 23042536 PMC4002216

[ref26] Li Y. Wang X. Bo K. Wang X. Tang B. Yang B.. (2007). Emergence of a highly pathogenic porcine reproductive and respiratory syndrome virus in the mid-eastern region of China. Vet. J. 174, 577–584. doi: 10.1016/j.tvjl.2007.07.032, PMID: 17869553

[ref27] Li X. Wu C. Chen N. Gu H. Yen A. Cao L.. (2016). PI3K/Akt/mTOR signaling pathway and targeted therapy for glioblastoma. Oncotarget 7, 33440–33450. doi: 10.18632/oncotarget.7961, PMID: 26967052 PMC5078108

[ref28] Li C. Zhao J. Li W. Xu H. Gong B. Sun Q.. (2024). Prevalence and genetic evolution of porcine reproductive and respiratory syndrome virus in commercial fattening pig farms in China. Porcine Health Manag. 10:5. doi: 10.1186/s40813-024-00356-y, PMID: 38254191 PMC10801985

[ref29] Liu J. Liu C. Xu Y. Yang Y. Li J. Dai A.. (2022). Molecular characteristics and pathogenicity of a novel recombinant porcine reproductive and respiratory syndrome virus strain from NADC30-, NADC34-, and JXA1-like strains that emerged in China. Microbiol. Spectr. 10:e0266722. doi: 10.1128/spectrum.02667-22, PMID: 36354339 PMC9769985

[ref30] Lunney J. K. Fang Y. Ladinig A. Chen N. Li Y. Rowland B.. (2016). Porcine reproductive and respiratory syndrome virus (PRRSV): pathogenesis and interaction with the immune system. Annu. Rev. Anim. Biosci. 4, 129–154. doi: 10.1146/annurev-animal-022114-111025 26646630

[ref31] Lupberger J. Duong F. H. Fofana I. Zona L. Xiao F. Thumann C.. (2013). Epidermal growth factor receptor signaling impairs the antiviral activity of interferon-alpha. Hepatology 58, 1225–1235. doi: 10.1002/hep.26404, PMID: 23519785

[ref32] Ma Y. J. Yang J. Fan X. L. Zhao H. B. Hu W. Li Z. P.. (2012). Cellular microRNA let-7c inhibits M1 protein expression of the H1N1 influenza A virus in infected human lung epithelial cells. J. Cell. Mol. Med. 16, 2539–2546. doi: 10.1111/j.1582-4934.2012.01572.x, PMID: 22452878 PMC3823446

[ref33] MacRae I. J. Ma E. Zhou M. Robinson C. V. Doudna J. A. (2008). In vitro reconstitution of the human RISC-loading complex. Proc. Natl. Acad. Sci. U. S. A. 105, 512–517. doi: 10.1073/pnas.0710869105, PMID: 18178619 PMC2206567

[ref34] Majed S. O. (2022). RNA sequencing-based total RNA profiling; the oncogenic MiR-191 identification as a novel biomarker for breast cancer. Cell. Mol. Biol. 68, 177–191. doi: 10.14715/cmb/2022.68.1.22 35809314

[ref35] Martinelli E. Morgillo F. Troiani T. Ciardiello F. (2017). Cancer resistance to therapies against the EGFR-RAS-RAF pathway: the role of MEK. Cancer Treat. Rev. 53, 61–69. doi: 10.1016/j.ctrv.2016.12.001, PMID: 28073102

[ref36] Mellios N. Huang H. S. Grigorenko A. Rogaev E. Akbarian S. (2008). A set of differentially expressed miRNAs, including miR-30a-5p, act as post-transcriptional inhibitors of BDNF in prefrontal cortex. Hum. Mol. Genet. 17, 3030–3042. doi: 10.1093/hmg/ddn201, PMID: 18632683 PMC2722882

[ref37] Mishra R. Kumar A. Ingle H. Kumar H. (2019). The interplay between viral-derived miRNAs and host immunity during infection. Front. Immunol. 10:3079. doi: 10.3389/fimmu.2019.03079, PMID: 32038626 PMC6989438

[ref38] Mitchell J. A. Aronson A. R. Mork J. G. Folk L. C. Humphrey S. M. Ward J. M. (2003). Gene indexing: characterization and analysis of NLM's GeneRIFs. AMIA Annu. Symp. Proc. 2003, 460–464. 14728215 PMC1480312

[ref39] Nagpal N. Ahmad H. M. Molparia B. Kulshreshtha R. (2013). MicroRNA-191, an estrogen-responsive microRNA, functions as an oncogenic regulator in human breast cancer. Carcinogenesis 34, 1889–1899. doi: 10.1093/carcin/bgt107 23542418

[ref40] Nagpal N. Kulshreshtha R. (2014). miR-191: an emerging player in disease biology. Front. Genet. 5:99. doi: 10.3389/fgene.2014.00099, PMID: 24795757 PMC4005961

[ref41] O'Brien J. Hayder H. Zayed Y. Peng C. (2018). Overview of MicroRNA biogenesis, mechanisms of actions, and circulation. Front. Endocrinol. 9:402. doi: 10.3389/fendo.2018.00402, PMID: 30123182 PMC6085463

[ref42] Pan Q. Xie Y. Zhang Y. Guo X. Wang J. Liu M.. (2024). EGFR core fucosylation, induced by hepatitis C virus, promotes TRIM40-mediated-RIG-I ubiquitination and suppresses interferon-I antiviral defenses. Nat. Commun. 15:652. doi: 10.1038/s41467-024-44960-6, PMID: 38253527 PMC10803816

[ref43] Pandita S. Verma A. Kumar N. (2023). Role of miRNAs in regulating virus replication. Anim. Gene 30:200162. doi: 10.1016/j.angen.2023.200162

[ref44] Pei Y. Lin C. Li H. Feng Z. (2023). Genetic background influences pig responses to porcine reproductive and respiratory syndrome virus. Front. Vet. Sci. 10:1289570. doi: 10.3389/fvets.2023.1289570, PMID: 37929286 PMC10623566

[ref45] Peng S. Wang J. Wei S. Li C. Zhou K. Hu J.. (2018). Endogenous cellular MicroRNAs mediate antiviral defense against influenza a virus. Mol. Ther. Nucleic Acids 10, 361–375. doi: 10.1016/j.omtn.2017.12.016, PMID: 29499948 PMC5862538

[ref46] Rahe M. C. Murtaugh M. P. (2017). Mechanisms of adaptive immunity to porcine reproductive and respiratory syndrome virus. Viruses 9:148. doi: 10.3390/v9060148, PMID: 28608816 PMC5490824

[ref47] Rathkjen P. H. Dall J. (2017). Control and eradication of porcine reproductive and respiratory syndrome virus type 2 using a modified-live type 2 vaccine in combination with a load, close, homogenise model: an area elimination study. Acta Vet. Scand. 59:4. doi: 10.1186/s13028-016-0270-z, PMID: 28057035 PMC5217557

[ref48] Sen N. Che X. Rajamani J. Zerboni L. Sung P. Ptacek J.. (2012). Signal transducer and activator of transcription 3 (STAT3) and survivin induction by varicella-zoster virus promote replication and skin pathogenesis. Proc. Natl. Acad. Sci. U. S. A. 109, 600–605. doi: 10.1073/pnas.1114232109, PMID: 22190485 PMC3258638

[ref49] Song L. Liu H. Gao S. Jiang W. Huang W. (2010). Cellular microRNAs inhibit replication of the H1N1 influenza a virus in infected cells. J. Virol. 84, 8849–8860. doi: 10.1128/jvi.00456-10, PMID: 20554777 PMC2919005

[ref50] Taguchi Y. H. Murakami Y. (2013). Principal component analysis based feature extraction approach to identify circulating microRNA biomarkers. PLoS One 8:e66714. doi: 10.1371/journal.pone.0066714, PMID: 23874370 PMC3715582

[ref51] Wang R. Wang X. Ni B. Huan C. C. Wu J. Q. Wen L. B.. (2016). Syndecan-4, a PRRSV attachment factor, mediates PRRSV entry through its interaction with EGFR. Biochem. Biophys. Res. Commun. 475, 230–237. doi: 10.1016/j.bbrc.2016.05.084, PMID: 27208778

[ref52] Wang C. Xue M. Wu P. Wang H. Liu Z. Wu G.. (2022). Coronavirus transmissible gastroenteritis virus antagonizes the antiviral effect of the microRNA miR-27b via the IRE1 pathway. Sci. China Life Sci. 65, 1413–1429. doi: 10.1007/s11427-021-1967-x, PMID: 34826094 PMC8617553

[ref53] Webster R. J. Giles K. M. Price K. J. Zhang P. M. Mattick J. S. Leedman P. J. (2009). Regulation of epidermal growth factor receptor signaling in human cancer cells by microRNA-7. J. Biol. Chem. 284, 5731–5741. doi: 10.1074/jbc.M804280200 19073608

[ref54] Wilczynska A. Bushell M. (2015). The complexity of miRNA-mediated repression. Cell Death Differ. 22, 22–33. doi: 10.1038/cdd.2014.112, PMID: 25190144 PMC4262769

[ref55] Xu P. Jia S. Wang K. Fan Z. Zheng H. Lv J.. (2020). MiR-140 inhibits classical swine fever virus replication by targeting Rab25 in swine umbilical vein endothelial cells. Virulence 11, 260–269. doi: 10.1080/21505594.2020.1735051, PMID: 32114898 PMC7051144

[ref56] Xu Y. Wang H. Zhang X. Zheng X. Zhu Y. Han H.. (2021). Highly pathogenic porcine reproductive and respiratory syndrome virus (HP-PRRSV) induces IL-6 production through TAK-1/JNK/AP-1 and TAK-1/NF-κB signaling pathways. Vet. Microbiol. 256:109061. doi: 10.1016/j.vetmic.2021.109061, PMID: 33836390

[ref57] Yang L. Wang R. Ma Z. Xiao Y. Nan Y. Wang Y.. (2017). Porcine reproductive and respiratory syndrome virus antagonizes JAK/STAT3 signaling via nsp5, which induces STAT3 degradation. J. Virol. 91:e02087-16. doi: 10.1128/jvi.02087-16, PMID: 27881658 PMC5244345

[ref58] Yang L. Xu J. Guo L. Guo T. Zhang L. Feng L.. (2018). Porcine epidemic diarrhea virus-induced epidermal growth factor receptor activation impairs the antiviral activity of type I interferon. J. Virol. 92:e02095-17. doi: 10.1128/jvi.02095-17, PMID: 29386292 PMC5874413

[ref59] Ye J. Guo R. Shi Y. Qi F. Guo C. Yang L. (2016). miR-155 regulated inflammation response by the SOCS1-STAT3-PDCD4 axis in atherogenesis. Mediat. Inflamm. 2016, 8060182–8060114. doi: 10.1155/2016/8060182, PMID: 27843203 PMC5098093

[ref60] Zhang Y. G. Chen H. W. Zhang H. X. Wang K. Su J. Chen Y. R.. (2022). EGFR activation impairs antiviral activity of interferon signaling in brain microvascular endothelial cells during Japanese encephalitis virus infection. Front. Microbiol. 13:894356. doi: 10.3389/fmicb.2022.894356, PMID: 35847084 PMC9279666

[ref61] Zhang Q. Guo X. K. Gao L. Huang C. Li N. Jia X.. (2014). MicroRNA-23 inhibits PRRSV replication by directly targeting PRRSV RNA and possibly by upregulating type I interferons. Virology 450-451, 182–195. doi: 10.1016/j.virol.2013.12.020, PMID: 24503081

[ref62] Zhang Q. Huang C. Yang Q. Gao L. Liu H. C. Tang J.. (2016). MicroRNA-30c modulates type I IFN responses to facilitate porcine reproductive and respiratory syndrome virus infection by targeting JAK1. J. Immunol. 196, 2272–2282. doi: 10.4049/jimmunol.1502006, PMID: 26826240

[ref63] Zhang J. Li A. Gu R. Tong Y. Cheng J. (2023). Role and regulatory mechanism of microRNA mediated neuroinflammation in neuronal system diseases. Front. Immunol. 14:1238930. doi: 10.3389/fimmu.2023.1238930, PMID: 37637999 PMC10457161

[ref64] Zhang H. Luo Q. He Y. Zheng Y. Sha H. Li G.. (2023). Research Progress on the development of porcine reproductive and respiratory syndrome vaccines. Vet. Sci. 10:491. doi: 10.3390/vetsci10080491, PMID: 37624278 PMC10459618

[ref65] Zhang L. Zhang L. Pan Y. Gao J. Xu Y. Li X.. (2021). Downregulation of miR-218 by porcine reproductive and respiratory syndrome virus facilitates viral replication via inhibition of type I interferon responses. J. Biol. Chem. 296:100683. doi: 10.1016/j.jbc.2021.100683, PMID: 33887325 PMC8131720

[ref66] Zheng Y. Yang Z. Jin C. Chen C. Wu N. (2021). Hsa-miR-191-5p inhibits replication of human immunodeficiency virus type 1 by downregulating the expression of NUP50. Arch. Virol. 166, 755–766. doi: 10.1007/s00705-020-04899-7, PMID: 33420627

[ref67] Zhou L. Yang H. (2010). Porcine reproductive and respiratory syndrome in China. Virus Res. 154, 31–37. doi: 10.1016/j.virusres.2010.07.016, PMID: 20659506

