# Supplementary material for: miR-191-5p suppresses PRRSV replication by targeting porcine EGFR to enhance interferon signaling
Source: Front Microbiol. 2024 Oct 14;15:1473504. doi: 10.3389/fmicb.2024.1473504 (PMC11514493; doi:10.3389/fmicb.2024.1473504)
Supplement: Supplementary file 4 [file Table_4.DOCX]

**Supplementary materials**

**Table S4.** The sequences constructed into plasmids

| Sequence Name | Sequence (5' - 3') |
| --- | --- |
| Pig EGFR mRNA 3UTR WT | ACGGTACTTACCCTCCATTGAGTGACCAGTGTTTTCTGATTATATGTGTTGGATTTACTTGTTTATTTTCCATTCCGTTGTTTTGAGATTCAGTGTGGTTTCCAGCAAGGGGAGATGGGACATCAGAACATTAGCCA |
| human EGFR mRNA 3UTR WT | ACGGTACTTACTCCCCACTGATGGACCAGTGGTTTCCAGTCATGAGCGTTAGACTGACTTGTTTGTCTTCCATTCCATTGTTTTGAAACTCAGTATGCTGCCCCTGTCTTGCTGTCATGAAATCAGCAAGAGA |
| Monkey EGFR mRNA 3UTR WT | ACGGTACTTACTCCCCACTGATTGACTAGTGGTTTCCAGTCGTGAGCGTTAGACTGACTTGTTTGTCTTCCATTCCATTGTTTTGAAACTCAGTATGCTGCCCCTGTCTTGCTGTCATGGAATCAGCAAGAGA |
| Pig EGFR mRNA 3UTR replaced by human | ACGGTACTTACCCTCCATTGAGTGACCAGTGTTTTCTGATTATATGTGTTGGATTGACTTGTTTGTCTTCCATTCCATTGTTTTGAGATTCAGTGTGGTTTCCAGCAAGGGGAGATGGGACATCAGAACATTAGCCA |
| Pig EGFR mRNA 3UTR mut | ACGGTACTTACCCTCCATTGAGTGACCAGTGTTTTCTGATTATATGTGTTGGATTGTTGCCTTACCTTTTATTTGTTCATTTTTGAGATTCAGTGTGGTTTCCAGCAAGGGGAGATGGGACATCAGAACATTAGCCA |
